# Impact of pyrolysis process conditions on the structure of biochar obtained from apple waste

**DOI:** 10.1038/s41598-024-61394-8

**Published:** 2024-05-07

**Authors:** Wioletta Barszcz, Monika Łożyńska, Jarosław Molenda

**Affiliations:** 1 Bioeconomy and Ecoinnovation Centre, Łukasiewicz Research Network – Institute for Sustainable Technologies, 26-600 Radom, Poland; 2 Faculty of Buildings Services, Hydro and Environmental Engineering, Warsaw University of Technology, 00-653 Warsaw, Poland

**Keywords:** Ecology, Environmental sciences

## Abstract

Biochar is an eco-friendly carbon material whose properties allow it to be used as a sorbent for wastewater treatment or soil remediation. The paper presents the results of research related to the pyrolysis process of apple waste after supercritical CO_2_ extraction with the simultaneous use of physical activation. The research assessed the influence of the temperature of the pyrolysis process and steam activation on the structural properties of the obtained biochar, i.e. specific surface, porous structure, and presence of functional groups. The results obtained confirmed that lower temperature pyrolysis produces biochar characterised by the presence of functional groups and ordered structure. On the other hand, high temperature pyrolysis with simultaneous steam activation determines microporosity and high values of the specific surface area. Taking into consideration pollutant sorption mechanisms (physical and chemical sorption), the obtained biochar materials can be used as sorbents in water and wastewater treatment.

## Introduction

Contemporary EU economic development strategies impose on the member states the obligation to implement the principles of circular economy. One of them is the production of waste-derived materials, which helps ensure closed-loop recycling. An example of a waste-derived material is biochar, which is an environmentally friendly product, whose properties allow for its wider application. Biochar is gradually replacing traditionally produced active carbon for wastewater treatment and soil remediation^1–4^. Plant-based biomass used for the production of biochar should come from waste, constitute no competition for human food, and require no multistage pretreatment^5–7^. Plant-based biomass can be divided into three main groups: agriculture residues, herbaceous biomass, and forest residues. Many types of biomass (e.g. fruit or vegetable pulps from the food industry, brewery spent grains, straw husk, sugarcane bagasse, switchgrass or wood chips) can be used to produce biochar^8–10^.

Biochar is obtained as a result of thermal conversion of plant-based biomass—a process which can employ different thermal decomposition methods (e.g. pyrolysis, gasification, torrefaction or hydrothermal carbonization)^11–13^, in addition to bio-oil and gas. The method most suitable for biochar production is pyrolysis, i.e. the heating of biomass in the absence of oxygen. There are three types of pyrolysis: (i) conventional/slow pyrolysis; (ii) fast pyrolysis; and (iii) ultra-fast/flash pyrolysis^14^. Each of them varies in terms of temperature, heating rate, and residence time^14–16^. Process parameters determine biochar’s structural properties. Researchers prove that high temperature, low pressure, and high heating rate of the pyrolysis process produce biochar with higher carbon content and large specific surface area^15,17^. Additionally, structural properties of biochar also depend on the type of the surface activation method used. Physical activation with steam, most commonly at 700–900 °C, helps expand the specific surface area of biochar and its microporous structure, as well as introduce functional groups on biochar surface^18,19^. Compared to chemical activation, physical activation reduces the number of unit steps necessary to perform, which reduces the production costs of biochar material. At the same time, it also makes it possible to reduce the use of chemical reagents in favor of more environmentally friendly components (in this case, water vapor). Adsorption of pollutants on the surface of physically activated biochar takes place mainly in multilayers using van der Waals forces. Such binding of pollutants is an easily reversible process compared to chemisorption. This aspect significantly facilitates the regeneration of the deposit, and thus has a positive impact on the economics of using such biochar materials in industry.

Given its specific properties, biochar can be used in different applications. Studies mainly focus on the use of biochar to remove pollutants from water or soil, i.e. cationic aromatic dyes (methylene violet, methylene blue, etc.), fertilizers (pesticides, herbicides, carbofurans, atrazine, etc.), antibiotics and medicines (ibuprofen, tetracyclines, sulfamethazine, etc.), polycyclic aromatic hydrocarbons (naphthalene, phenanthrene, nitrotoluene, etc.) or volatile organic compounds (benzene, furan, butanol, etc.)^20–23^. Apart from organic pollutants, biochar can also remove inorganic compounds like non-biodegradable heavy metals. Experiments in this regard concern the removal of the following ions: Pb^2+^, Cu^2+^, Zn^2+^, Cr^3+^^24–26^. Biochar is also studied for adsorption of compounds in industrial and municipal wastewaters, i.e.: NO_3_^−^, NH_4_^+^ or H_2_S^27,28^.

The removal of pollutants with the use of biochar can happen during physical or chemical sorption. These mechanisms require different biochar properties. In physical sorption, a developed specific surface area of carbon materials is crucial. On the other hand, in chemical sorption, biochar should be characterised by the presence of functional groups capable of binding pollutants^24,29^. Therefore, the selection of a suitable carbon material with proper characteristics is of key importance when managing biochar as a pollutant sorbent.

The main aims of the paper are focused on the determination of the impact of pyrolysis conditions (i.e. temperature, steam activation) on structural properties of carbon material obtained from apple waste, as well as on analysis of the development of the specific surface area, porosity, surface functional groups, biochar structure.

## Materials and methods

### Materials

Biochar was produced from apple biomass, a residue from the supercritical CO_2_ extraction of antioxidants used in the production of cosmetics. The biomass used had low moisture content (< 10%).

### Pyrolysis

To produce biochar, 100 g of apple waste were pyrolysed. Pyrolysis was conducted with (BA600, BA700, BA800) or without (B300, B700) steam activation to determine the impact of pyrolysis condition on biochar properties. A Czylok FCF-V12RM muffle furnace was used. The cascade temperature control programs used are presented in Table 1.

**Table. 1:** Temperature program of the apple waste pyrolysis process.

Temperature program of pyrolysis			B300	B700	BA600	BA700	BA800
Step 1	Temp. [°C]		200	200	200	200	200
	Time [min]		15	15	15	15	15
	Rate [°C/min]		12	12	12	12	12
	Hold [min]		15	15	15	15	15
Step 2	Temp. [°C]		250	650	550	650	750
	Time [min]		15	15	15	15	15
	Rate [°C/min]		3	30	23	30	37
	Hold [min]		15	15	15	15	15
Step 3	Temp. [°C]		300	700	600	700	800
	Time [min]		15	15	15	15	15
	Rate [°C/min]		3	3	3	3	3
	Hold [min]		15	15	15	15	15
Activation H_2_O_(g)_	Temp. [°C]		–	–	600	700	800
	Time of processes [min]	Start			60	60	60
		End			105	105	105

Additionally, steam activation was carried out for samples marked BA600, BA700, and BA800. According to literature data, is carried out at high temperatures of up to 900 °C for 45–60 min^19,29^. For this reason, the production of biochar at 300 °C with steam activation was abandoned. Whereas, the physical activation performed with steam at temperatures in the range of 600–800 °C was intended to show the changes that occur in biochar when the temperature of the pyrolysis process increases with simultaneous activation. Activation started at 60 min of pyrolysis and lasted 45 min. Steam flow rate was 8.0 dm^3^/min. After pyrolysis, the samples were left in the furnace for 12 h to cool to room temperature. Carbon dioxide (flow rate of 5.0 dm^3^/min) was used as a protective gas during heating and cooling processes.

### Structural properties of biochar produced

The biochar produced was characterised in terms of its structural properties, i.e. surface morphology, porosity, presence of functional groups, and graphitization degree. To this end, ground biochar samples (fraction < 2 mm) were used.

### SEM/EDS microscopy

Surface morphology and elemental composition of the biochar produced was analysed using a Hitachi SU-70 electron microscope with an X-ray microanalyzer (EDS). The analyses were conducted under the following conditions: magnification: × 500 and × 1000; accelerating voltage: 15 kV; inclination angle 30°; and vacuum 10^−8^ Pa.

### Physical adsorption

Porosity was measured based on N_2_ adsorption isotherms (77 K) determined with a Quantachrome AUTOSORB IQ analyzer. Prior to analysis, the samples were degassed under vacuum (10^−7^ bar) at 350 °C for 12 h. The specific surface area, volume, and average size of pores, as well as the volume and surface area of the micropores were calculated from the data obtained in the physisorption process. Specific surface area was determined using the multipoint Brunauer–Emmett–Teller (BET) method. The volume and average size of pores were calculated using the quenched solid density functional theory (QSDFT) for characterisation of mico- and mesoporous carbons. The volume and surface of micropores was calculated with the t-plot method.

### FTIR spectroscopy

Functional groups on the surface of biochar were identified with an FTIR 6200 Fourier spectroscope. Infrared spectra were recorded using a Pike diamond ATR detector. For spectral measurements, a TGS detector was used in the spectral range of 4000–650 cm^−1^ and resolution of 4 cm^−1^.

### Raman spectroscopy

The crystal structure of the biochar obtained was analysed using a Jasco NRS-5100 Raman spectroscope. Raman spectra were taken at room temperature using laser excitation at 532 nm for an exposure time of 210 s. The spectra were recorded for wavenumbers ranging from 300 to 3200 cm^−1^ and resolution of 3.62 cm^−1^.

## Results and discussion

### SEM/EDS microscopy

**Figure 1:**
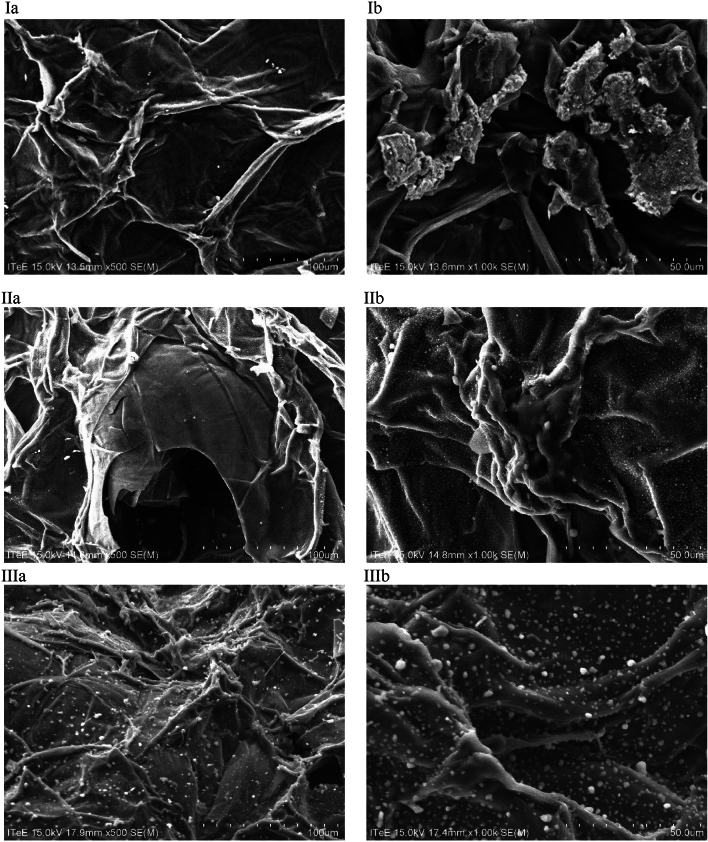

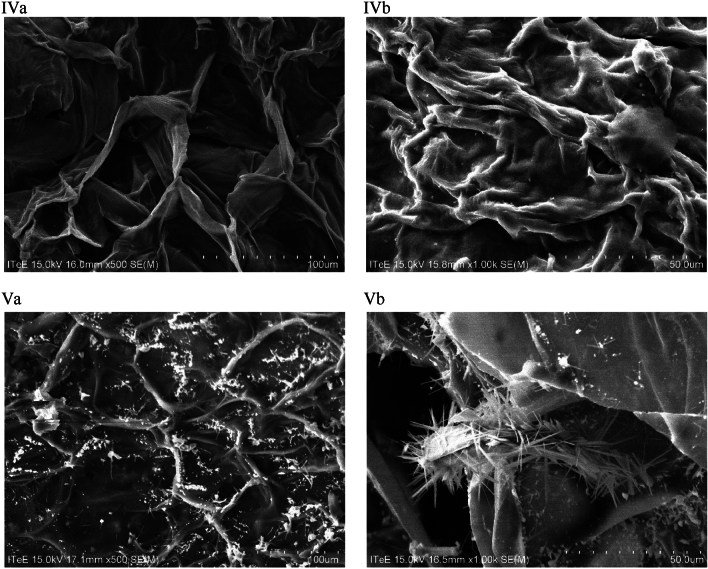
SEM images of biochar taken after pyrolysis at magnification (**a**) × 500 and (**b**) × 1000: (I) B300; (II) B700; (III) BA600; (IV) BA700; (V) BA800.

The biochar produced was also characterised in terms of its structural properties, i.e. surface morphology, porosity, presence of functional groups, and crystal structure. Figure 1 presents the images of biochar surface morphology taken with an electron microscope at magnification × 500 and × 1000.

The analysis of the microscopic images shows that the biochar produced has an irregular, fibrous, and three-dimensional structure. The EDS analysis presented in Table 2 indicates that in addition to the main components (i.e. 57–80% carbon and 9–30% oxygen, depending on the pyrolysis process), biochar also contained nitrogen (1–10%), sodium (approx. 2%), potassium (approx. 5%), and chlorine (approx. 2%)—these elements were probably present in the biomass pyrolysed or could constitute residues of synthetic fertilizers used in horticulture.

**Table 2:** EDS analysis for produced biochars.

Element	B300	B700	BA600	BA700	BA800
Carbon [%_mas._]	57.64	71.28	61.11	83.05	55.12
Oxygen [%_mas._]	30.14	12.89	22.25	9.16	22.84
Nitrogen [%_mas._]	5.89	4.19	7.82	1.31	3.86
Sodium [%_mas._]	0	3.34	2.96	1.29	6.18
Chloride [%_mas._]	1.36	2.48	1.35	1.38	4.52
Potassium [%_mas._]	4.97	5.82	4.51	3.81	7.48

Different forms of precipitates (depending on the pyrolysis process) could be observed on all biochar samples analysed. In the case of samples B700, BA600, and BA700, precipitates had the form of microspheres. As for samples B300 and BA800, they had the form of particle agglomerates and needles, respectively. The EDS analysis of these structures showed that their elemental composition differed from that of biochar. A higher content of sodium (approx. 8%), chlorine (approx. 22%), and potassium (approx. 29%) was observed, which suggests that precipitates are crystalised sodium and potassium chloride salts. Pyrolysis at 800 °C melts sodium and potassium salts (KCl and NaCl melting points: 775 and 801 °C, respectively). During cooling, these salts start to crystalise, which explains the change in the structure of crystallites in the BA800 biochar sample compared to other biochar samples analysed.

### Physical adsorption

Nitrogen 77 K adsorption and desorption isotherms were determined using a Quntachrome sorption analyzer to measure porosity of the biochar produced. Based on the physisorption data, the specific surface area, volume, and average size of pores, as well as the volume and surface area of the micropores were determined with the following methods: MBET, QSDFT, and t-plot. The obtained results are presented in Table 3.

**Table 3:** Comparison of textural parameters of pure and steam-activated biochar.

Biochar sample	Isotherm type	S_MBET_ [m^2^/g]	D_m_ [nm]	V_t_ [cm^3^/g]	V_mic_ [cm^3^/g]	S_mic_ [m^2^/g]	S_mic_/S_MBET_ [%]
B300	III	14.4	4.323	0.015	ND	ND	ND
B700	I(b)	314.7	1.299	0.138	0.112	287.0	91.20
BA600	I(a)	408.3	0.889	0.236	0.130	323.9	79.33
BA700	I(a)	647.0	0.753	0.250	0.202	587.9	90.87
BA800	I(a)	1 120.0	0.753	0.502	0.288	911.1	81.35

*S*_*MBET*_ specific surface area; *D*_*m*_ average pore width; *V*_*t*_ total volume of pores, *V*_*mic*_ volume of micropores; *S*_*mic*_ micropore surface; *ND* not detected.

To determine the isotherm types, the IUPAC classification was applied^30^. All biochar samples but B300 were characterised by isotherm I typical of microporous materials. Such an isotherm is characterised by an intensive deposition of adsorbent at low p/p_0_ pressure ratios. Biochar obtained as a result of steam activation is characterised by adsorption isotherm type I(a) typical of materials with narrow micropores (< 1 nm). Isotherm obtained for sample B700 is isotherm type I(b) characteristic of materials containing wider micropores and narrow mesopores (< 2.5 nm). On the other hand, isotherm obtained for sample B300 is isotherm type III characteristic of nonporous or macroporous materials. The matching of isotherm types is consistent with the results of the average pore size obtained using the QSDFT method. Steam-activated biochar samples had pores less than 1 nm wide (BA600: 0.889 nm, BA700: 0.753 nm, and BA800 0.753 nm), and B700: 1.299 nm. This confirms that the biochar samples analysed are microporous materials^31^. The widest pores (4.323 nm) were observed in biochar pyrolysed at 300 °C. Given its small specific surface area, it can be stated that this biochar is a low-porous material.

The obtained results of the specific surface area calculated with the MBET method show big differences depending on the type of pyrolysis. Both pyrolysis temperature and steam activation of biochar play an important role. Increasing pyrolysis temperature enables increasing specific surface area of biochar by progressively degrading organic matter and removing pore-blocking substances^32,33^. Specific surface area can also be increased through physical activation of biochar materials^29^. Activation was conducted in parallel with pyrolysis during the last 45 min of this process. Steam temperature depended on the final temperature of pyrolysis (Table 1). The higher the steam activation temperature, the larger the specific surface area of the material, which is caused by quicker removal of carbon from the surface and evaporation of volatile substances contained in the material. This has also been confirmed by other authors^34–37^. For example, Rajapaksha et al.^37^ compared properties of pure and steam-activated biochar obtained from tea waste at 300 and 700 °C and found that at a lower steam temperature the specific surface area grew six times compared to the initial value and 76 times at higher steam temperature.

Pure biochar (i.e. not activated with steam) is characterised by a relatively small specific surface area, which gradually grows when the pyrolysis temperature is increased (14.4 m^2^/g for B300, and 314.7 m^2^/g for B700). This relationship was also observed in the case of steam-activated biochar (408 m^2^/g for BA600, 647 m^2^/g for BA700, and 1120 m^2^/g for BA800 (Table 3). As specific surface area grows, the total pore volume also increases. This relationship has a very strong linear correlation, which is confirmed by the Pearson correlation coefficients of 0.98 for the correlation between S_MBET_ and V_t_ and 0.99 for the correlation between S_mic_ and V_mic_. Simultaneous increase in the specific surface area and pyrolysis temperature has also been confirmed by other researchers^38–41^. Jin et al. (2020) produced biochar from grape waste (from various stages of wine production) during pyrolysis conducted at 300, 500, and 700 °C (time: 2 h, heating rate: 10 °C/min). They found that different temperature and biomass composition results in the production of biochar with different BET surface. Biochar from grapes before fermentation has specific surface area between 1.03 and 4.10 m^2^/g, while biochar from grapes after enzymatic hydrolysis—between 82.2 and 485 m^2^/g; however, in both cases, specific surface area increases in parallel with the increase in pyrolysis temperature^38^. Elnour et al. (2019) analysed the impact of pyrolysis temperature on microstructural properties of biochar obtained from date palm waste. The analysis of specific surface area showed that biochar produced at 700 °C has S_BET_ = 249.130 m^2^/g, i.e. 122 times higher than in the case of biochar obtained at 300 °C (S_BET_ = 2.04 m^2^/g)^42^. Han et al., who produced biochar from switchgrass, hardwood, and softwood, came to similar conclusions. By increasing the temperature and introducing steam activation to the pyrolysis process, they produced biochar with a specific surface area greater by an average of 3.5 times^43^. Bouchelta et al.^44^ analysed the impact of temperature and steam activation on textural properties of biochar from date waste and found that introducing steam as an activator at peak temperatures increases microporosity of the material, which is probably due to the elimination of volatile substances produced during pyrolysis, as a result of which centres active on the biochar surface open and new pores are formed in the structure.

**Figure 2:**
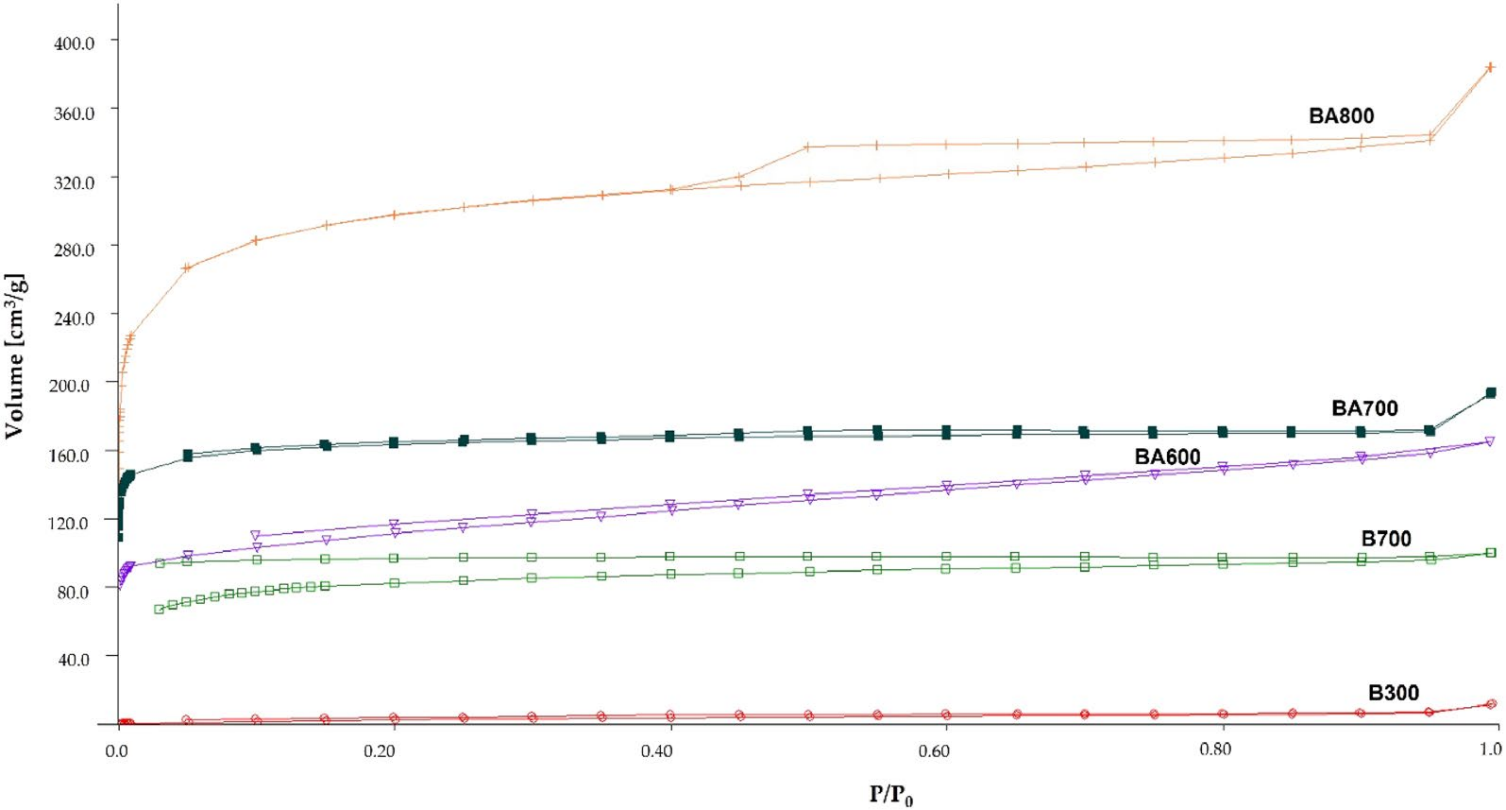
N_2_ adsorption isotherms at 77 K for pure (B300, B700) and steam-activated (BA600, BA700, and BA800) biochar.

Based on the percentage relationship between the microporous area and specific surface area (S_mic_/S_MBET_), it was also observed that the main factor affecting biochar microporosity is the temperature of the pyrolysis process (Table 3). Although B300 can be considered a nonporous material, when the pyrolysis temperature was increased, the biochar’s specific surface area became dominated by micropores (91%). In the case of activated biochar, the authors observed that there were more micropores in the specific surface area of the BA700 biochar compared to BA600 (90% and 70%, respectively). Further increase in the pyrolysis temperature to 800 °C decreased the micropore area to 81%. This could be due to the breakdown of micropores and, thus, an increase in the number of mesopores at high physical activation temperatures^45,46^. This is also confirmed by the H4 hysteresis loop observed for biochar BA800 (Fig. 2). This isotherm type indicates the micro-mesoporous nature of the sample. Such hysteresis is characterised by a rapid descend of the desorption curve at the p/p_0_ value characteristic of the adsorption isotherm of 77 K nitrogen of approx. 0.4–0.5. This has been confirmed by other authors as well. Zhou et al. (2018) analysed the impact of activation time and steam activation temperature on the porosity and specific surface area of tea-based biochar. They found that the creation of mesopores at high activation temperatures (700–900 °C) was caused by the degradation of the micropore walls due to too rapid reactions between steam and carbon, involving only the outer surface of the biomass. Similar relationship was observed for the correlation between the steam activation time and temperature—the longer the activation time and the higher the activation temperature, the worse porosity of biochar^47^.

Comparing the S_mic_/S_MBET_ relationship of B700 (91.2%) and BA700 (90.9%) samples, it can be concluded that the temperature of the pyrolysis process, regardless of the activation used, is the main factor determining the number of micropores in the biochar structure.

### FTIR spectroscopy

**Figure 3:**
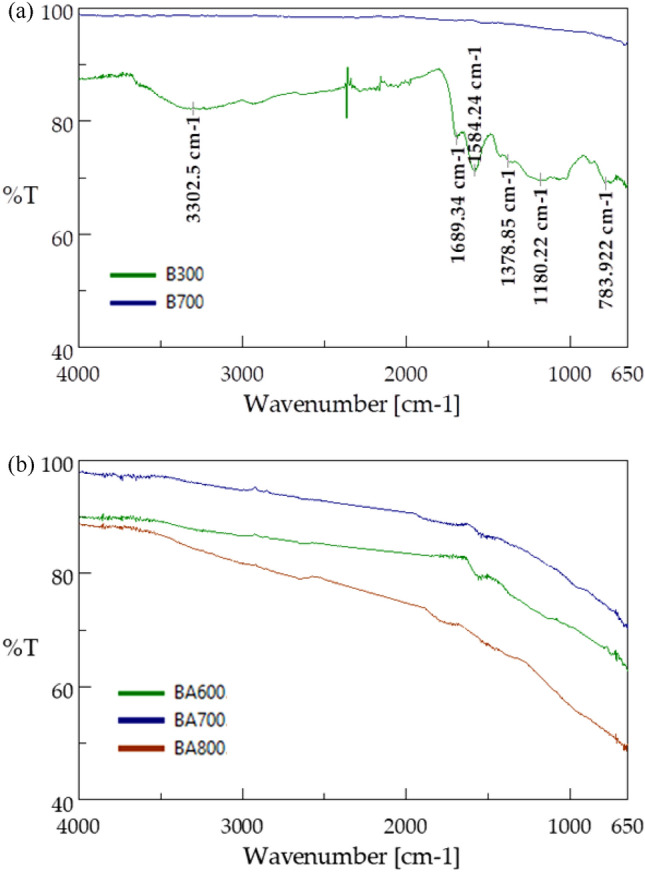
FTIR spectra of: (**a**) pure biochar (B300 and B700); (**b**) steam-activated biochar (BA600, BA700, and BA800).

The authors analysed the presence of functional groups on biochar surface to determine the impact of pyrolysis on biochar characteristics. The obtained FTIR spectra are presented in Fig. 3.

The analysis of the spectra shows that bands are present only in the case of the B300 biochar, which suggests the presence of functional groups on its surface. Higher pyrolysis temperatures (> 600 °C) cause the disappearance of functional groups containing hydrogen and oxygen as a result of deoxygenation and dehydration of the biomass^35^. The B300 biochar has a band located at 3302 cm^−1^ and corresponding to stretching vibrations of the –O–H bond^48–50^. At initial pyrolysis stages, at a temperature between 200 and 500 °C, initial degradation takes place, during which cellulose and hemicellulose are pyrolysed. At this stage, biochar is formed as a result of inter- and intramolecular reactions. Cellulose degradation reactions include decarboxylation, dehydrogenation, deoxygenation, and aromatization. At 300 °C, hydrogen bonds break down, resulting in a dehydration reaction, and as the temperature increases, hemicellulose transforms and surface functional groups (mainly hydroxyl and methoxyl groups) are reduced^51,52^.

In the case of the B300 biochar, peaks indicative of the presence of valence vibrations of the C=O group at 1689 cm^−1^ (a band probably originating from aromatic cyclocarbonyl) were also observed^53^. The presence of the 1584 cm^−1^ band indicates the presence of stretching vibrations of the C=C bonds in the aromatic ring^54^, while the 1378 cm^−1^ band corresponds to deformation vibrations of –O–H from alcohol or phenol derivatives^55^. The stretching vibration at 1180 cm^−1^ comes from the C–O bond. In the 783 cm^−1^ region, the observed band comes from bending vibrations of the = C–H group from polycyclic aromatic hydrocarbons^25^.

The analysis of the other spectra obtained indicates the absence of functional groups in all samples produced at temperatures above 600 °C (Fig. 3). Thus, it can be concluded that the presence of functional groups on biochar surface is strongly dependent on the temperature of the pyrolysis process. At higher temperatures, the number of oxygen, carboxyl or phenolic functional groups decreases as a result of their degradation. Banik et al. also showed that as the temperature increases, the number of oxygen heteroatoms from hydroxyl groups decreases due to the predominance of condensed polycyclic aromatic structures^17,29^.

### Raman spectroscopy

**Figure 4:**
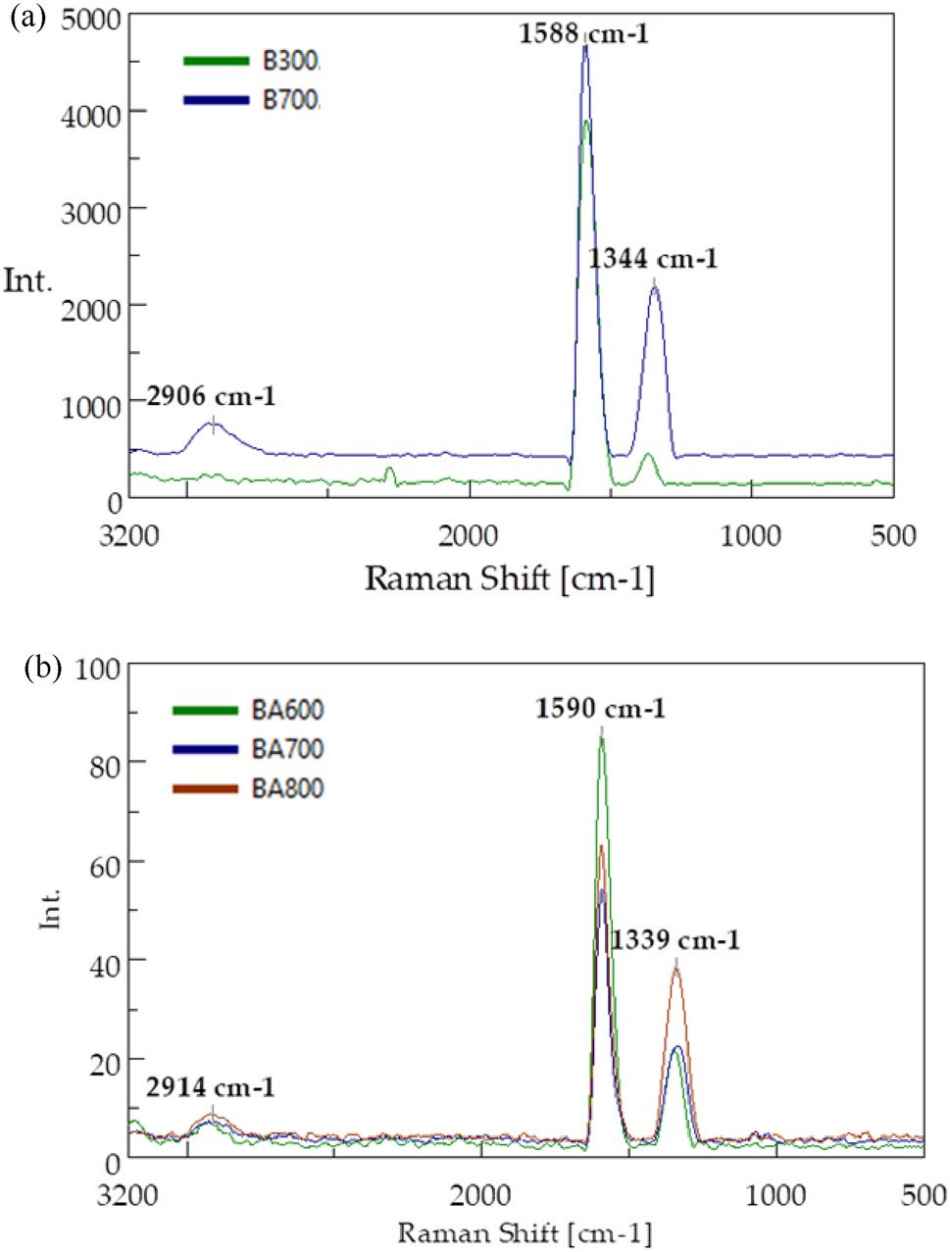
Raman spectra of: (**a**) pure biochar (B300 and B700); (**b**) steam-activated biochar (BA600, BA700, and BA800).

The biochar produced was characterised in terms of the order of the structure with Raman spectroscopy. The obtained spectra are presented in Fig. 4.

Two bands characteristic of carbons were detected in all types of the biochar produced: G-band at wavenumber 1592 cm^−1^ and D-band at 1350 cm^−1^. The G-band specifying the graphene structure originates from stretching vibrations of carbon bond pairs corresponding to stretching vibrations with symmetry ${E}_{2g}^{2}$ caused by the vibration of sp^2^ carbon bond pairs (C=C). Therefore, the biochar produced was characterised by the presence of ring structures, which may be associated with the presence of ordered carbon structures. The D-band, also known as the defect band, characterises the degree of amorphousness of carbon structures. It corresponds to A_1g_ and is caused by stretching vibrations of carbon C–C bond pairs and condensed aromatic rings. The emerging peaks in the range between 3400 and 2650 cm^−1^ are mainly attributed to aliphatic and aromatic *v*CH stretching vibrations resulting from combustion-induced structural modifications^56^. Determination of the I_D_/I_G_ intensity ratio is an important aspect of assessing the order of the carbon structure. The lower the I_D_/I_G_ value, the higher the degree of carbon graphitization. The relationships calculated for the biochar produced are presented in Table 4.

**Table 4:** Raman intensity ratios of biochar samples.

Sample of biochar	Non-activation		Steam activation		
	B300	B700	BA600	BA700	BA800
$\frac{{I}_{D}}{{I}_{G}}$	0.06	0.50	0.25	0.55	0.82

The analysis of the I_D_/I_G_ ratio found that biochar produced at the lowest temperature (i.e. 300 °C) had the most ordered structure. For this biochar, the I_D_/I_G_ ratio was 0.06. As a result of a higher pyrolysis temperature, the biochar structure became disordered, as indicated by higher values of the I_D_/I_G_ ratios ranging between 0.25 and 0.82. Biochar produced at 800 °C after steam activation had the most disordered structure and highest I_D_/I_G_ ratio = 0.82. Comparing the I_D_/I_G_ of biochar produced at 700 °C not activated with steam (I_D_/I_G_ = 0.50) and steam activated (I_D_/I_G_ = 0.55), it can be assumed that steam activation has no significant impact on structure order. The increase in the I_D_/I_G_ ratio parallel to the increase in the pyrolysis temperature is attributed to the evolution of gas species, including CH_4_, CO_2_, CO and H_2_O, formed during pyrolysis^57^. The impact of higher temperature of pyrolysis on the degree of amorphousness of biochar has also been confirmed by other authors. Chatterjee et al. also observed an increase in I_D_/I_G_ ratios as a result of an increase in the temperature of pyrolysis of different biomass types. For biochar obtained from miscanthus pyrolysed at 500 °C, the ratio is 0.65, and at 800 °C – 0.88. In the case of corn stover, this ratio grew from 0.58 at 500 °C to 0.88 at 800 °C and for sugarcane bagasse from 0.59 to 1.01^57^. Amdani et al. analysed beech- and pine tree-derived biochar and observed that the I_D_/I_G_ ratio increased by 17% and 30%, respectively, when the pyrolysis temperature increased from 200 to 350 °C^58^.

## Conclusions

Based on the obtained results it can be concluded that pyrolysis with steam activation enables production of microporous biochar with a developed specific surface area. The authors found that (i) elevated pyrolysis temperature increases the specific surface area and microporosity of biochar and that (ii) simultaneous steam activation further develops their specific surface area. The temperature of the pyrolysis process has a major impact on the presence of functional groups and the order of the biochar structure. Low temperature (300 °C) determines the creation of functional groups and a high degree of biochar graphitization. High temperature, on the other hand, causes the disappearance of functional groups and an amorphous structure of biochar. The conclusions were formulated based on the SEM/EDS, FTIR, Raman, and physisorption analyses of the biochar structure.

## Data Availability

The datasets used and analysed during the current study available from the corresponding author on reasonable request. All data generated or analysed during this study are included in this published article.
